# Supplementary material for: RNA N6-Methyladenosine Affects Copper-Induced Oxidative Stress Response in Arabidopsis thaliana
Source: Noncoding RNA. 2024 Jan 19;10(1):8. doi: 10.3390/ncrna10010008 (PMC10892094; doi:10.3390/ncrna10010008)
Supplement: Supplementary file 1 [file ncrna-10-00008-s001.zip › ncrna-2600546-supplementary.pdf]

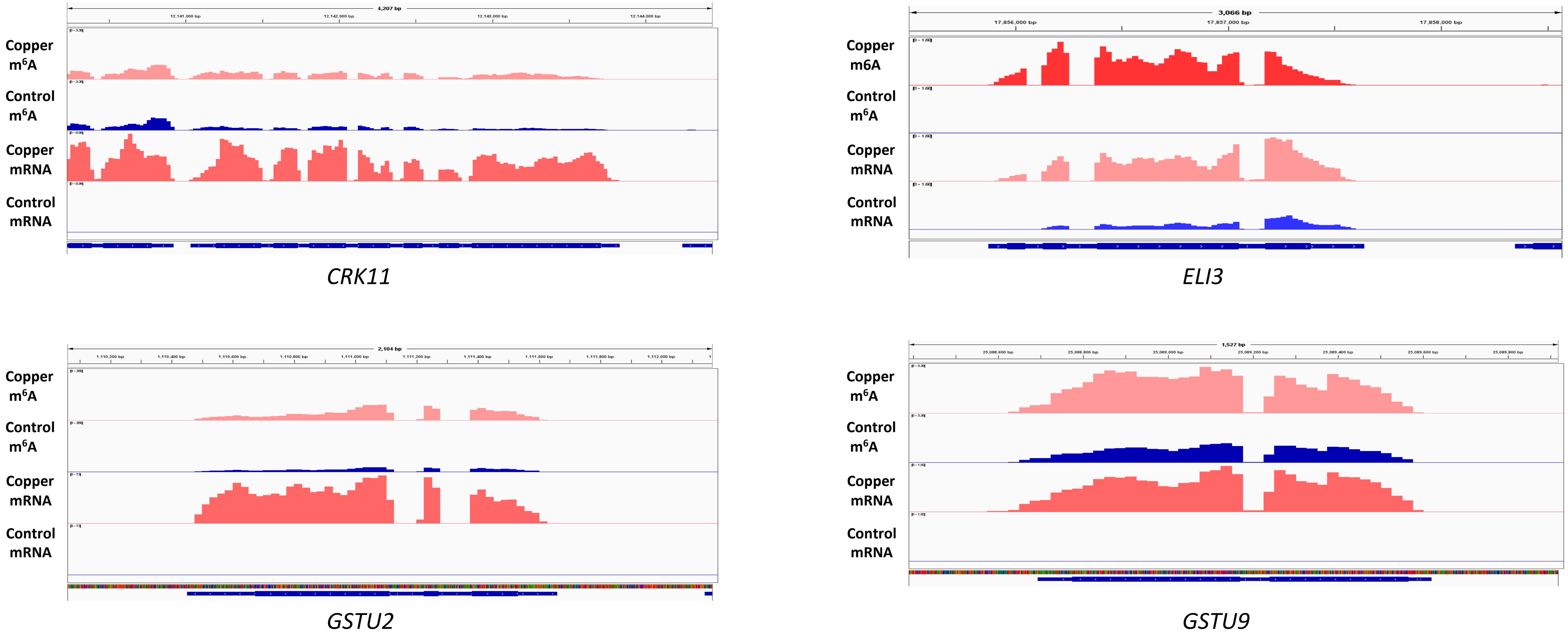

Supplemental Figure S1: Example transcripts displaying copper-induced oxidative stress-specific m6A increases that correspond with increases in overall mRNA abundance of those transcripts. The transcripts shown here are *CRK11*, *ELI3*, *GSTU2*, and *GSTU9* as indicated.

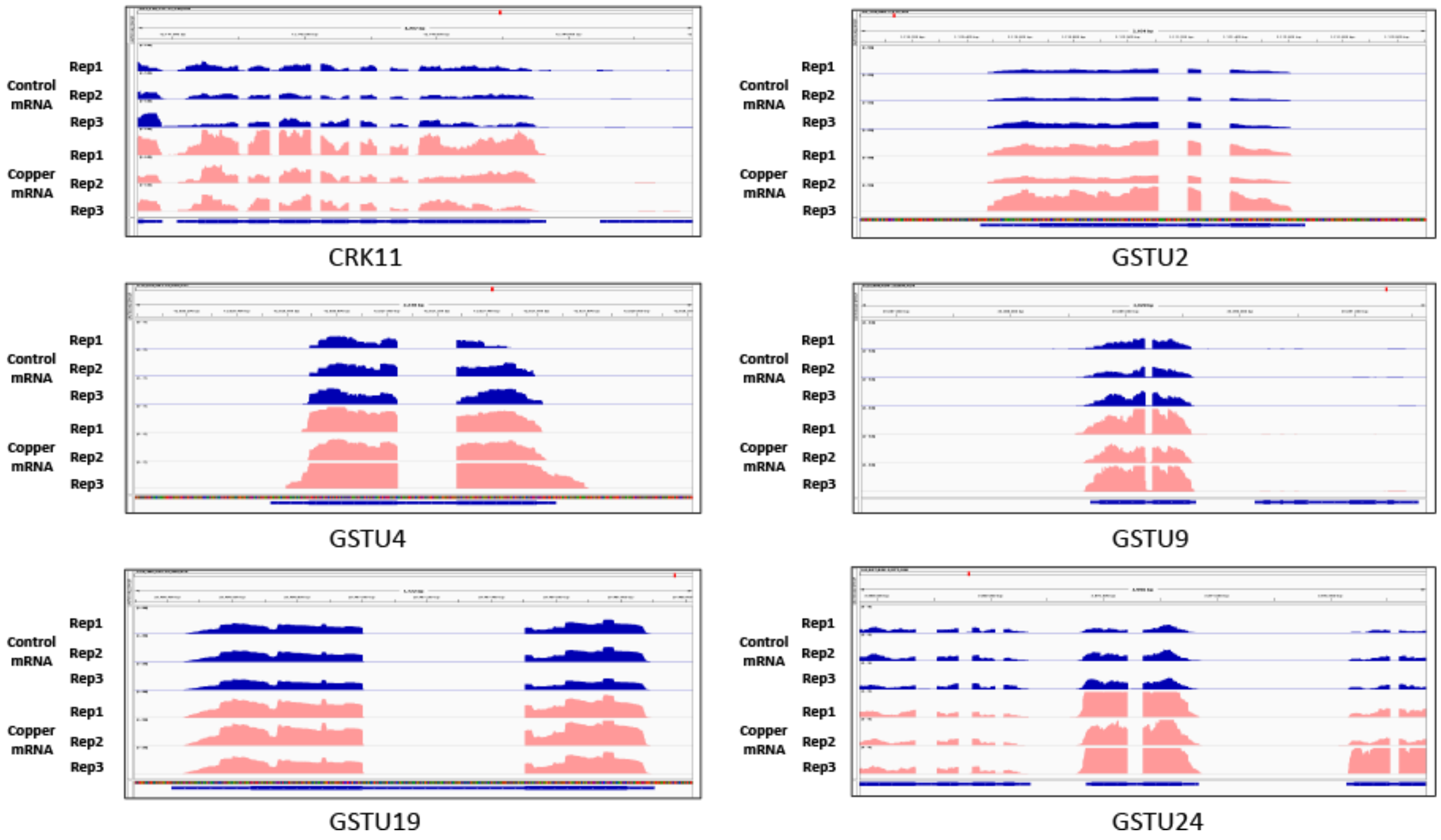

Supplemental Figure S2: Example transcripts displaying copper-induced oxidative stress-specific increases in overall mRNA abundance of those transcripts. The transcripts shown here are *CRK11*, *GSTU2*, *GSTU4*, *GSTU9*, *GSTU19*, and *GSTU24* as indicated.
